# Brachycephalic obstructive airway syndrome: much more than a surgical problem

**DOI:** 10.1080/01652176.2022.2145621

**Published:** 2022-11-15

**Authors:** Stefanie Mitze, Vanessa R. Barrs, Julia A. Beatty, Stefan Hobi, Paweł M. Bęczkowski

**Affiliations:** a Zoetis UK Ltd, Leatherhead, UK; b Department of Veterinary Clinical Sciences, Jockey Club College of Veterinary Medicine and Life Sciences, City University of Hong Kong, Hong Kong, SAR China; c Centre for Animal Health and Welfare, Jockey Club College of Veterinary Medicine and Life Sciences, City University of Hong Kong, Hong Kong, SAR China

**Keywords:** Canine, bulldog, brachycephaly, airway obstruction, surgery, ethical, welfare

## Abstract

Brachycephalic obstructive airway syndrome (BOAS) is a chronic, lifelong, debilitating, primarily obstructive airway disease which adversely affects the quality of life of many popular dog breeds. Respiratory restriction in bulldog breeds, pugs and Boston terriers frequently co-exist with pathologies of the gastrointestinal tract. In addition, many brachycephalic dogs that appear clinically normal are, in fact suffering from chronic hypoxia and its systemic consequences. Concurrent gastroesophageal reflux-associated conditions, sleep disorders and systemic hypertension further impact the welfare of affected dogs. Acceptance of BOAS and associated clinical signs as being ‘normal for the breed’ is common amongst owners. While surgical correction of the upper airway is the mainstay of treatment, the provision of subsequent, frequently lifelong medical management is equally important for the maintenance of an acceptable quality of life, at least for some affected patients. Here we review the current knowledge concerning brachycephaly, combine it with shared clinical experience in the management of this debilitating condition, and discuss ethical considerations and the responsibility of veterinarians to contribute public education and to support appropriate breed standards for animals under our care.

## 1. Introduction

Brachycephaly, derived from the ancient Greek words ‘brakhu’ (short) and ‘cephalos’ (head), is the terminology for a foreshortened skull (Ita and Rizvi 2021). This trait has been strongly selected for in some of the most popular canine breeds of the current era including pugs, French bulldogs, British bulldogs, and Boston terriers (Roedler et al. 2013; Liu et al. 2017; Siedenburg and Dupré 2021). The extreme brachycephalic conformation of these breeds is represented by their high cephalic index (CI), which is calculated by dividing skull width by skull length, expressed as a percentage (Georgevsky et al. 2014). The CI of wolves, the ancestors of modern-day dogs, is ∼50 (McGreevy et al. 2013). While the CI has been preserved in dolichocephalic breeds such as the Doberman Pinscher (CI ∼44), in some extremely brachycephalic breeds, such as pugs, the CI has been shown to be as high as ∼ 99 (McGreevy et al. 2013).

Brachycephaly was considered advantageous for many centuries in dogs used for bullbaiting, a blood sport that was practiced up to the nineteenth century (Dawson 1964; Gray 1980; Kalof and Taylor 2007). The strong and shortened jaws made it easier for dogs to bite the bull in the head and snout (Dawson 1964). Although bullbaiting has died-off, brachycephalic dog breeds are increasingly popular due to their ‘snub-nose’ or ‘flat-faced’ appearance, an infantile, anthropomorphic aesthetic feature that owners may find endearing (Uvarov 1985; Roedler et al. 2013; Liu et al. 2017; Ekenstedt et al. 2020). Other features that have contributed to the soaring popularity of extreme brachycephalic breeds in the last two decades include their small stature and low levels of aggression which make them popular family pets in urban settings (Kenny et al. 2022; O’Neill et al. 2022).

Selective breeding for extreme brachycephaly has given rise to a conformation-related respiratory disorder, known as brachycephalic obstructive airway syndrome (BOAS) (Dupré and Heidenreich 2016; Ladlow et al. 2018). Affected dogs have deformation of the upper respiratory tract due to shortening of the skull without a proportional decrease in the soft-tissues of the head, as well as other structural abnormalities involving the lower respiratory tract (Knecht 1979; Ekenstedt et al. 2020).

Diagnosis is often delayed due to a limited recognition of the problem by owners and the lack of an absolute clinical gold standard, making BOAS and its systemic consequences frequently underdiagnosed (Roedler et al. 2013; Ekenstedt et al. 2020; Kenny et al. 2022). In a recent survey of owners of brachycephalic and non- brachycephalic dogs, common clinical signs of BOAS such as snoring, snorting, and loud breathing were considered normal for brachycephalic breeds in ∼75% of respondents (Kenny et al. 2022).

Diagnosis of BOAS is based upon pre- and post-exercise clinical signs, clinical examination, and advanced imaging techniques such as endoscopy, computed tomography (CT) and magnetic resonance imaging (MRI) which are used for identification and grading (Roedler et al. 2013; Liu et al. 2015; 2016; 2017; Kim et al. 2019). Studies utilising plethysmography, a novel non-invasive technique that facilitates an objective assessment of respiratory function, suggest that both dogs with BOAS, and dogs that have undergone surgical corrective surgery for BOAS remain affected by systemic consequences of the disease (Liu et al. 2015; 2016). Extreme brachycephaly is associated with a chronic hypoxia that has similar consequences to human obstructive sleep apnoea (OSA) (O’Neill et al. 2015; Erjavec et al. 2021).

The objective of this review is to critically appraise current knowledge concerning BOAS in dogs, focussing on pathologies which have the greatest impact on the quality of life and on medical management, which is as important as corrective surgery. We highlight that canine BOAS should be managed as a systemic disease, not just as a pathology of the head and upper airways. In addition, we discuss ethical concerns and responsibilities of veterinarians to address the health and welfare impacts of extreme brachycephaly.

## 2. Abnormalities of the airways

BOAS is composed of primary and secondary airway abnormalities (Roedler et al. 2013; Liu et al. 2017; Ekenstedt et al. 2020). The most prominent primary anatomical malformations include stenotic nares, aberrant nasopharyngeal turbinates, an elongated and thickened soft palate, macroglossia, and sometimes a hypoplastic trachea (Wykes 1991; Hendricks 1992). Increased airway resistance imposed by these anatomical abnormalities leads to chronic barotrauma, development of secondary airway changes and a self-perpetuating, vicious cycle of respiratory pathologies (Ekenstedt et al. 2020).

### 2.1. Primary abnormalities

Stenotic nares are defined as narrowed external nostrils, and reduced diameter of nasal vestibules that lead into the nasal cavity (Roedler et al. 2013; Liu et al. 2017; Ekenstedt et al. 2020). This varying and abnormal narrowing, classified as mild, moderate, or severe, forces dogs to breathe with an open mouth (Ekenstedt et al. 2020). Early rhinoplasty of brachycephalic puppies is recommended to reduce the likelihood of secondary consequences of upper airway obstruction (Aron and Crowe 1985) (Table 1).

**Table 1.** Treatment options for brachycephalic obstructive airway syndrome (BOAS)-related pathologies.

Pathology	Surgical Treatments	Medical treatments	Comments
Stenotic nares	Vertical (Harvey 1982), horizontal (Leonard 1956), lateral (Monnet and Kudnig 2003) wedge alarplasty Alapexy (Ellison 2004) Punch resection alaplasty (Trostel and Frankel 2010) CO_2_ laser ablation (Mendes Junior et al. 2021)	–	Rhinoplasty is recommended for puppies of 12–16 weeks of age to reduce the likelihood of secondary consequences of upper airway obstruction (Aron and Crowe 1985)
Elongated soft palate	Staphylectomy (electroscalpel (Knecht 1979), radiofrequency (Elkins 2005), CO_2_ laser (Clark and Sinibaldi 1994), bipolar sealing device (Davidson et al. 2001) Folded flap palatoplasty (Findji and Dupre 2008) H-pharyngoplasty (Carabalona et al. 2022)	–	CO_2_ laser is the fastest technique (Davidson et al. 2001). Dogs <2 years of age treated with staphylectomy had better outcome than older dogs (Harvey 1982) H-pharyngoplasty may be suitable for most severely affected dogs (Carabalona et al. 2022).
Everted tonsils	Tonsillectomy (Fasanella et al. 2010)	–	Rarely performed due to unclear benefit (Poncet et al. 2005)
Everted laryngeal saccules	Sacculectomy (Harvey 1982)	–	Clinical improvement post sacculectomy only in 40% of cases (Lorinson et al. 1997)
Laryngeal collapse	Arytenoidectomy (Wykes 1991) Partial laryngectomy, laryngeal tieback, tracheostomy (Johnston and Tobias 2017)	Weight loss (Thomason et al. 2016) Exercise restriction (Welcome 2008)	Laryngeal collapse is challenging to manage with aspiration pneumonia being most common complication (Harvey 1982) Laryngotomy and tracheostomy are salvage procedures (Johnston and Tobias 2017)
Middle ear effusion (MEE)	Tympanostomy tube placement (Guerin et al. 2015)	Local and systemic corticosteroids, amitriptyline, antihistamines, N-acetylcysteine (Milne et al. 2020)	MEE can be subclinical, or associated with vestibular signs (Guerin et al. 2015)
Hiatal hernia (HH)	Oesophagopexy (Johnston and Tobias 2017) Gastropexy (Johnston and Tobias 2017)	Proton pump inhibitor, cisapride, feeding management (Hwang et al. 2017)	–
Reflux oesophagitis	–	Proton pump inhibitor, cisapride, sucralfate (Kook 2021)	–
Systemic hypertension	–	Calcium channel blocker, ACEI, ARB (Acierno et al. 2018)	Evaluate for concurrent causes of secondary hypertension (Acierno et al. 2018)
Hypercoagulability	–	Antiplatelet therapy (Thomason et al. 2016)	–

Mucosal hypertrophy of the turbinates and mucosal contact points (MCPs) within the nasal cavity, lead to reduced ventilation of sinuses, airflow resistance, nasal blockage, reduction in olfactory sense and predisposition to chronic lymphoplasmacytic rhinitis (Auger et al. 2016; Gianella et al. 2020). In humans, MCPs are associated with nasal blockage and altered sense of smell, while resultant stimulation of the trigeminal nerve is linked to facial pain defined as rhinogenic contact point headache (RCPH) (Mantia et al. 2018). Whether similar pain occurs in dogs is not known. Canine MCPs can be resected surgically, however relapse is common due to regrowth (Schuenemann and Oechtering 2014).

An elongated and thickened soft palate will frequently overlap the epiglottis and is responsible for the distinctive gagging, and retching (Roedler et al. 2013; Crosse et al. 2015). The rostral thickness of the soft palate was previously attributed to the muscle hypertrophy. However, based on the histological examination of affected tissue, mucous gland hyperplasia and oedema accompanied by an apparent reduction in muscle mass due to necrosis and degeneration predominate (Pichetto et al. 2011; Crosse et al. 2015).

The progressive increased airway resistance and associated pressure changes exacerbate the thickening and elongation of the soft palate over time (Pichetto et al. 2011; Crosse et al. 2015). These findings are similar to those described in the soft palates of humans with obstructive sleep apnoea (OSA) (Pichetto et al. 2011; Crosse et al. 2015). They are observed even in low grade adult brachycephalic dogs, but are not evident in brachycephalic neonates (Pichetto et al. 2011; Crosse et al. 2015; Pichetto et al. 2015). Redundant soft palate tissue can be corrected surgically, with electrocautery or a CO_2_ laser (Tamburro et al. 2019; Conte et al. 2022) (Table 1).

An enlarged tongue (macroglossia) relative to the widened and shortened head contributes further to the dorsal displacement of the soft palate, and increases the turbulent airflow in the nasopharynx. Surgical correction of macroglossia has been described in humans with OSA, but not in brachycephalic dogs (Jones et al. 2020; Siedenburg and Dupré 2021).

Tracheal hypoplasia occurs frequently in English bulldogs and screw-tailed brachycephalic breeds (Komsta et al. 2019; Regier et al. 2020). It is characterised by small and rigid tracheal cartilage rings and a shortened or absent dorsal tracheal membrane. Tracheal hypoplasia is not always associated with clinical signs. However, it is recognised as a negative prognostic indicator in patients with concurrent bronchopneumonia (Siedenburg and Dupré 2021). In young growing dogs an improvement of the tracheal diameter is possible (Clarke et al. 2011), but it does not increase in adult dogs following BOAS surgery (Regier et al. 2020). Tracheal hypoplasia can be accompanied by thoracic wall and spinal deformities such as kyphosis and scoliosis (Ryan et al. 2017; Komsta et al. 2019). Rare congenital chest wall anomalies such as pectus excavatum (PE) and pectus carinatum (PC) are characterised by the dorsal and ventral displacement of the sternum and associated ribs, respectively (Ryan et al. 2017; Komsta et al. 2019). Those anatomical abnormalities are more common in certain brachycephalic dog breeds and contribute to decreased thoracic wall flexibility, air-trapping, atelectasis, reduced lung compliance and in some cases, respiratory failure (Ryan et al. 2017; Komsta et al. 2019).

### 2.2. Secondary abnormalities

Enlarged or protruding palatine tonsils are a result of the chronic airway pressure changes, irritation, and inflammation (Köhler et al. 2022). Their enlargement has been linked with respiratory infection and decreased exercise tolerance (Montague et al. 2002). Everted palatine tonsils can be treated by tonsillectomy, although the benefit is unclear (Turkki et al. 2022).

As the disease progresses due to the long-term negative pressure gradients, many brachycephalic dogs develop pharyngeal collapse. This is a partial or complete narrowing of the pharynx due to dorsal dislocation of the soft palate, ventral displacement of the dorsal pharyngeal wall, or a combination thereof (Pollard et al. 2018; Hara et al. 2020).

Eversion of the laryngeal saccules and laryngeal collapse during BOAS is due to cartilage fatigue and chondromalacia, both reducing the laryngeal stiffness (Tokunaga et al. 2020). Repetitive micro-trauma and abnormal mucosal contact lead to secondary inflammation and development of laryngeal contact granulomas on the vocal cords, epiglottic cysts and excessive aryepiglottic folds (Sarran et al. 2020). Everted laryngeal saccules and selected cases of laryngeal collapse can be addressed surgically (Table 1).

Bronchial abnormalities occur in over 85% of dogs with BOAS (De Lorenzi et al. 2009). The location and severity are variable, however collapse of the left main and left cranial dorsal subsegmental bronchus are most common (Bernaerts et al. 2010; Reinero and Masseau 2021). Bronchial collapse has its biggest impact during expiration, which is usually a passive process. However in BOAS, upper airway resistance leads to an increased intraluminal pressure gradient during inspiration, resulting in a forced active expiration and consequent airway collapse. Bronchial collapse occurs at an early age, progresses to bronchial stenosis and over time may lead to development of pulmonary hypertension and its sequelae (De Lorenzi et al. 2009; Yoon et al. 2020).

Brachycephaly confers an increased risk of aspiration pneumonia and pneumonitis, emphasising the importance of thoracic imaging in the presurgical work-up of these patients (Ree et al. 2016; Darcy et al. 2018). Treatment is frequently complicated by gastroesophageal reflux and recurrence of aspiration events (Table 1).

## 3. Middle ear effusions

Middle ear effusions (MEE) sometimes occur in brachycephalic dogs and lead to an increased mucus accumulation within the tympanic bulla (Paterson 2017; Milne et al. 2020; Schuenemann et al. 2022). The secretion is typically viscous and sterile (Milne et al. 2020). The goblet cells of the tympanic bulla continuously produce mucus, which flows through the Eustachian tube into the nasopharynx (Nuutinen et al. 1983). In brachycephalic dogs, reduced nasopharyngeal width adversely affects clearance of the mucus (Paterson 2017; Milne et al. 2020). In addition, increased mucus production and pressure differences may also play a role (Stern-Bertholtz et al. 2003; Hayes et al. 2010). MEE frequently are an incidental finding but can also be linked to head and neck pain, unnatural neck carriage, abnormal vocalisation, pruritus, impaired hearing, head shaking, vestibular signs, and lethargy (Schuenemann et al. 2022). Treatment of symptomatic cases involves placement of tympanostomy tubes, repeated video-otoscopic middle ear flushes and supportive medical treatment with corticosteroids, amitriptyline, antihistamines, or mucolytic N-acetylcysteine (Corfield et al. 2008; Guerin et al. 2015; Paterson 2017) (Table 1). Since an allergic reaction is suspected as part of the pathogenesis, local and systemic corticosteroids and antihistamines improve opening of the Eustachian tube (Stern-Bertholtz et al. 2003; Paterson 2017). Amitriptyline is used to treat neuropathic pain, but also due to its ability to disturb bacterial biofilm and antimicrobial properties (Cashmore et al. 2009; May et al. 2016). Systemic mucolytics are reported to reduce relapse (Hayes et al. 2010; Paterson 2017).

## 4. Abnormalities of the gastrointestinal tract

Prognathism, frequently present as underbite, although not anatomically normal, is considered a standard in brachycephalic dogs (Regalado Ibarra and Legendre 2019; O'Neill et al. 2021). This malocclusion leads to traumatic contact of the mandibular incisors with the hard palate, subsequent pulpitis and focal palatitis (Regalado Ibarra and Legendre 2019). Other abnormalities of the oral cavity include dental crowding, tooth rotation, unerupted teeth, prominent palatine rugae, periodontal disease and consequent systemic bacteraemia (Regalado Ibarra and Legendre 2019; O'Neill et al. 2021; Harvey 2022).

The respiratory and gastrointestinal tract share overlapping mechanisms required for breathing and swallowing and can pathologically affect each other (Grobman 2021; Luciani et al. 2022). The prevalence of gastrointestinal disease in brachycephalic dogs presenting for respiratory signs is as high as 97% (Poncet et al. 2005 2006; Kaye et al. 2018; Freiche and German 2021). The most common clinical signs are regurgitation, vomiting and dysphagia (Roedler et al. 2013; Freiche and German 2021).

Congenital or acquired hiatal hernia (HH), frequently represents an additional finding for dogs presenting predominately for BOAS surgery. HH defined as prolapse of abdominal organs through the oesophageal hiatus into the mediastinum is exacerbated in brachycephalic dogs by an abnormal intrathoracic and intra-oesophageal pressure (Reeve et al. 2017; Broux et al. 2018; Eivers et al. 2019). Herniation of the stomach leads to chronic regurgitation and predisposes to oesophagitis, gastric dilatation–volvulus and aspiration pneumonia (Mayhew et al. 2021). Severe cases of HH can be treated surgically with gastropexy or oesophagopexy (Lorinson and Bright 1998; Johnston and Tobias 2017; Mayhew et al. 2017). Medical management is focussed on the reduction of gastroesophageal reflux events (Sivacolundhu et al. 2002) (Table 1).

Oesophageal motility disorders are common in brachycephalic dogs, and include prolonged oesophageal transit time (Reeve et al. 2017), and prolonged secondary peristaltic waves (Eivers et al. 2019). These variations predispose to gastroesophageal reflux disease (GERD) frequently documented in brachycephalic dogs (Poncet et al. 2005 2006; Reeve et al. 2017; Kaye et al. 2018; Eivers et al. 2019). GERD is defined as the pathological reflux of gastric or duodenal contents with resultant oesophagitis, oesophageal ulceration, and in some cases oesophageal strictures (Freiche and German 2021). The pathophysiology is multifactorial. Incompetence of the lower oesophageal sphincter (LES), obesity and increased abdominal pressure are major contributors (Mönkemüller et al. 2012; Kempf et al. 2014).

Signs of GERD include lip smacking, ptyalism, extension of head and neck while swallowing, retching, vomiting, regurgitation, grass ingestion, surface licking, dysphagia, nocturnal restlessness, and anorexia (Poncet et al. 2005, 2006; Reeve et al. 2017; Eivers et al. 2019). Affected dogs frequently bring up frothy sputum during exercise or excitement (Kempf et al. 2014).

In humans, the clinical presentation of GERD is classified into: 1) oesophageal, and 2) extraoesophageal syndromes (Vakil et al. 2006).

Oesophageal syndromes are subdivided into a) symptomatic syndromes, such as typical reflux syndrome and reflux chest pain syndrome, and b) syndromes with oesophageal injury such as oesophagitis, stricture, Barrett’s oesophagus, and oesophageal adenocarcinoma.

Extraoesophageal syndromes are subdivided into: a) established and b) proposed associations. Established associations include reflux-cough, reflux-laryngitis, reflux-asthma and reflux-dental erosion syndromes. Proposed associations include sinusitis, idiopathic pulmonary fibrosis, pharyngitis, and otitis media.

To the best of our knowledge, no studies have investigated similar associations in brachycephalic dogs. However, one study suggests a link between GERD and idiopathic rhinitis (Gianella et al. 2020).

The severity and frequency of reflux in brachycephalic dogs can be reduced medically (Table 1). Therapy includes a frequent, small bolus feeding regime and administration of a prokinetic and a proton pump inhibitor, and in cases of suspected oesophagitis an addition of sucralfate (Grobman 2021; Kook 2021). Cisapride is superior to metoclopramide in increasing the LES pressure, and esomeprazole is superior to omeprazole in increasing gastric pH (Hwang et al. 2017). However, consequences of long-term treatment with proton pump inhibitors include potential small intestinal dysbiosis, atrophic gastritis, hypomagnesemia, hypocobalaminemia, and hypocalcaemia (Katz 2018).

The correct diagnosis of gastrointestinal diseases merits special consideration, because brachycephalic dogs are commonly affected by concurrent gastrointestinal disorder, such as protein-losing enteropathy in pugs (Freiche and German 2021), granulomatous colitis in boxers, mastiffs, and French bulldogs (Manchester et al. 2013).

## 5. Sleep disorders in brachycephalic dogs

Canine BOAS share similarities to obstructive sleep apnoea (OSA) in humans and therefore brachycephalic dogs can serve as a valuable models of human disease (Hendricks et al. 1987; Veasey et al. 1999). Abnormal sleep behaviour in brachycephalic dogs is common and linked to abnormal sleep disordered breathing (SDB) (Hendricks et al. 1987; Veasey et al. 1999). It encompasses a wide range of abnormalities such as sleeping with an open mouth in sitting or elevated positions, sleep fragmentation, sleep apnoea, sleep arousal or complete inability to sleep with consequent daytime hypersomnolence (Foldvary-Schaefer and Waters 2017). Owners often do not perceive sleeping problems as restrictive for their dog’s welfare compared to the exercise intolerance caused by BOAS (Barker et al. 2021).

OSA in humans, is a complex pathological process that occurs as a result of a muscular relaxation and subsequent intermittent partial or complete blockage of the pharynx (Gianella et al. 2019). This phenomenon is also described in English bulldogs and worsens during REM phase, particularly in older, obese dogs (Hendricks et al. 1987).

In humans and dogs, the cycle of chronic intermittent hypoxaemia and reoxygenation, resembles repeated ischaemia and reperfusion events (Gianella et al. 2019; Facin et al. 2020). This oxidative stress leads to the formation of reactive nitrogen and oxygen species, inflammation, and dysfunction of vascular endothelium and has been linked with atherosclerosis and cardiovascular disease (Vakil et al. 2006).

Human OSA patients and brachycephalic dogs share similar clinicopathological abnormalities such as decreased arterial partial pressure of oxygen (PaO_2_), increased arterial partial pressure of carbon dioxide (PaCO_2_), increased haematocrit (HCT), hyperglycaemia, hyperlipidaemia, and increased levels of C-reactive protein (CRP) (Mellema and Hoareau 2014; Hoareau and Mellema 2015; Gianella et al. 2019). In human OSA patients, these findings are suggestive of activation of a chronic stress response due to hypoxia, insulin resistance, altered hepatic lipid metabolism and systemic inflammation. The same mechanism is suspected in brachycephalic dogs (Hendricks et al. 1987; Gianella et al. 2019; Barker et al. 2021; Jawa et al. 2021). In fact, the antioxidant enzyme, superoxide dismutase (SOD) levels are increasing in the sera of brachycephalic dogs after corrective surgery. This paradoxical increase is suggestive of an improved postoperative oxygenation, making SOD a potential biomarker of oxidative stress (Lavie 2015; Erjavec et al. 2021).

### 5.1. Hypoxaemia, hypertension, and hypercoagulability

In human OSA cases, chronic hypoxaemia leads to liver hypoxia, inflammation, fibrosis, and portal hypertension (Weingarten et al. 2012; Chou et al. 2015; Benotti et al. 2016; Jin et al. 2018; Jawa et al. 2021). The liver injury observed in human OSA patients is described as non-alcoholic fatty liver disease (NAFLD) (Jawa et al. 2021). It has been demonstrated that dogs with BOAS have increased tissue stiffness of the liver and spleen, and therefore it is possible that similar pathologies occur (Facin et al. 2020).

Dogs with BOAS have significant higher systolic, mean and diastolic arterial blood pressure in comparison to a dolichocephalic and mesocephalic control group (Hoareau et al. 2012; Mellema and Hoareau 2014). Although an exact underlying mechanism is unknown, increased sympathetic activity, metabolic dysfunction, endothelial damage and hypomagnesemia are thought to play a role (Hoareau et al. 2012; Mellema and Hoareau 2014). Hypertensive brachycephalic dogs need to be thoroughly evaluated for concurrent causes of secondary hypertension before institution of treatment (Acierno et al. 2018) (Table 1).

Persistent chronic inflammation, increased platelet activation and delayed fibrinolysis promote the hypercoagulable state and predispose brachycephalic dogs to thromboembolic disease (Hoareau and Mellema 2015; Crane et al. 2017). Therapeutic considerations include antiplatelet agents (Table 1).

## 6. Ethical concerns, responsibilities of veterinarians and preventive strategies

The graduation oaths of veterinarians from most countries contain statements affirming an obligation to practice not just for the benefit of an individual animal, but for the benefit of animal health and welfare in general. In addition, the global professional organisation for veterinarians, the World Small Animal Veterinary Association (WSAVA) also asks members to swear an oath that includes the statement ‘As a global veterinarian, I will use my knowledge and skills for the benefit of our society through the protection of animal welfare and health and the prevention and relief of animal suffering’. Thus, while it is beholden upon veterinarians to manage extreme brachycephaly on a case-by-case basis at the individual level of the patient and client, it can be argued that veterinarians also have a duty-of-care for broader engagement on the issue, for example with breeders, the community, and policymakers (Fawcett et al. 2018). Even in the absence of membership of such organisations, veterinarians have an ethical responsibility to prevent and minimise the negative health and welfare impacts of extreme brachycephaly on the basis that affected animals do not enjoy all the five freedoms of animal welfare under human control (freedom from hunger and thirst, freedom from discomfort, freedom from pain, injury and disease, freedom from fear and distress, and freedom to express normal behaviour) (Webster 2016).

Professional organisations such as the British Veterinary Association (BVA) have made online tools available for veterinarians, including a 10-point plan to improve the health and welfare of brachycephalic dogs and to promote responsible ownership (Atkin 2020). At the patient level, the plan includes offering new dog owners pre-purchase consultations to identify potential health problems in dogs with extreme brachycephalic conformation, performing exercise tolerance and functional grading as part of an annual health assessment and providing strong advice against breeding of dogs with BOAS or that require conformation altering surgery. At the community level, the plan includes development and observation of a practice communication strategy and mechanisms to support local breed clubs and representatives.

In addition to the efforts of professional veterinary associations including one organisation dedicated to the issue (Vets Against Brachycephalism), there are many cross-organisational initiatives such as the Brachycephalic Working Group (www.ukbwg.org.uk) and the Love is Blind campaign of the Australian Veterinary Association and the RSPCA (www.ava.com.au/love-is-blind/) that aim to prevent conformation-related health problems and reduce the demand for brachycephalic dogs. However, despite the traction that the issue of extreme brachycephaly has gained because of engagement by the veterinary profession, charitable animal organisations and extensive media coverage, there is a continued upsurge in brachycephalic dog ownership (Farnworth 2022; Loeb and Williams 2022). Other strategies may be necessary to make an impact to improve the health and welfare of brachycephalic dogs.

Legislative approach has been started in Europe to enact more rapid change (Commission européenne 2020). The Netherlands has a pro-healthy brachycephalic breeding legislation and clearly defined criteria for the enforcement of the article 3.4 of the Animal Keepers Decree which rules against breeding of companion animals that is detrimental to their welfare (Hagen 2019). A similar approach has been enforced in Norway and although currently under appeal, based on the two cases of an English bulldog and a Cavalier King Charles Spaniel, a court ruling in Oslo declared that breeding of dogs with inherited diseases breaches the country’s Animal Welfare Act (Norway Government 2009).

## 7. Conclusions

Consequences of airway obstruction and reflux-associated conditions in brachycephalic dogs are progressive and can eventually lead to multiorgan dysfunction. The evidence that brachycephalic dogs, including those without overt respiratory signs, are systemically affected emphasises the importance of early recognition. So far, the most promising, objective diagnostic method is whole-body barometric plethysmography which can help to avoid delays in clinical decision making. Serum SOD offers a promising surrogate marker and its utility in the clinical setting warrants further research.

Corrective surgery for BOAS, although associated with potential serious complications is often recommended (Ree et al. 2016; Hughes et al. 2018; Fenner et al. 2020; Mayhew et al. 2021). Marked post-operative improvement is reported in 30–90% of cases (Lorinson et al. 1997; Torrez and Hunt 2006; Riecks et al. 2007; De Lorenzi et al. 2009; Pohl et al. 2016). However, direct comparison of the surgical success rate is challenging due to lack of an objective, standardised scoring system (Packer and Tivers 2015). Adjuvant medical therapy targeting oesophageal and gastrointestinal problems, is frequently employed as it has been shown to decrease the risk of postsurgical complications and to substantially improve the prognosis and long-term outcome in over 70% of brachycephalic dogs (Poncet et al. 2006). Weight loss, exercise restriction and environmental modifications, are equally important in the management of brachycephalic dogs, regardless of the medical and surgical treatments (Gossellin et al. 2007; Manens et al. 2012; Packer and Tivers 2015).

Although the surgical and non-surgical management of BOAS patients is our duty in the clinical setting, it is indisputable that in many cases a fully successful outcome is unachievable (Packer and Tivers 2015). Therefore, our biggest responsibility is to work together with all stakeholders to advise and enact policy change and guide public education and new breed standards for animals under our care.
